# Widespread Circulation of Flaviviruses in Horses and Birds in Northeastern Spain (Catalonia) between 2010 and 2019

**DOI:** 10.3390/v13122404

**Published:** 2021-11-30

**Authors:** Sebastian Napp, Francisco Llorente, Cécile Beck, Eduard Jose-Cunilleras, Mercè Soler, Lola Pailler-García, Rayane Amaral, Pilar Aguilera-Sepúlveda, Maria Pifarré, Rafael Molina-López, Elena Obón, Olga Nicolás, Sylvie Lecollinet, Miguel Ángel Jiménez-Clavero, Núria Busquets

**Affiliations:** 1 IRTA, Animal Health Research Centre (CReSA IRTA-UAB), 08193 Bellaterra, Spain; lola.pailler@irta.cat; 2 Centro de Investigación en Sanidad Animal (CISA), Instituto de Investigación y Tecnología Agraria y Alimentaria (INIA-CSIC), 28130 Valdeolmos, Spain; dgracia@inia.es (F.L.); aguilera.pilar@inia.es (P.A.-S.); majimenez@inia.es (M.Á.J.-C.); 3 UMR 1161 Virology, ANSES, INRAE, ENVA, ANSES Animal Health Laboratory, EURL for Equine Diseases, 94704 Maisons-Alfort, France; cecile.beck@anses.fr (C.B.); rayane.amaralmoraes@anses.fr (R.A.); sylvie.lecollinet@cirad.fr (S.L.); 4 Departament de Medicina i Cirurgia Animals, Facultat de Veterinària, Universitat Autònoma de Barcelona, 08193 Bellaterra, Spain; eduard.jose.cunilleras@uab.cat; 5 Servei Medicina Interna Equina, Fundació Hospital Clínic Veterinari (UAB), 08193 Bellaterra, Spain; 6 Servei de Prevenció en Salut Animal, Departament d’Acció Climàtica, Alimentació i Agenda Rural (DACC), 08007 Barcelona, Spain; merce.soler@gencat.cat; 7 Centre de Fauna dels Aiguamolls de l’Empordà, Àrea de Gestió Ambiental Servei de Fauna i Flora, Forestal Catalana, 17486 Castelló d’Empúries, Spain; maria.pifarre@gencat.cat; 8 Centre de Fauna de Torreferrussa, Àrea de Gestió Ambiental Servei de Fauna i Flora, Forestal Catalana, 08130 Santa Perpètua de Mogoda, Spain; rafael.molina@gencat.cat (R.M.-L.); elena.obon@gencat.cat (E.O.); 9 Centre de Fauna de Vallcalent, Àrea de Gestió Ambiental Servei de Fauna i Flora, Forestal Catalana, 25199 Lleida, Spain; olga.nicolas@gmail.com; 10 Parc Natural de l’Alt Pirineu, Àrea de Gestió Ambiental Servei de Fauna i Flora, Forestal Catalana, 25595 Llavorsí, Spain; 11 CIBER of Epidemiology and Public Health (CIBERESP), 28029 Madrid, Spain

**Keywords:** West Nile virus, Usutu virus, Bagaza virus, tick-borne encephalitis virus, flaviviruses, Spain

## Abstract

The surveillance for West Nile virus (WNV) in Catalonia (northeastern Spain) has consistently detected flaviviruses not identified as WNV. With the aim of characterizing the flaviviruses circulating in Catalonia, serum samples from birds and horses collected between 2010 and 2019 and positive by panflavivirus competition ELISA (cELISA) were analyzed by microneutralization test (MNT) against different flaviviruses. A third of the samples tested were inconclusive by MNT, highlighting the limitations of current diagnostic techniques. Our results evidenced the widespread circulation of flaviviruses, in particular WNV, but also Usutu virus (USUV), and suggest that chicken and horses could serve as sentinels for both viruses. In several regions, WNV and USUV overlapped, but no significant geographical aggregation was observed. Bagaza virus (BAGV) was not detected in birds, while positivity to tick-borne encephalitis virus (TBEV) was sporadically detected in horses although no endemic foci were observed. So far, no human infections by WNV, USUV, or TBEV have been reported in Catalonia. However, these zoonotic flaviviruses need to be kept under surveillance, ideally within a One Health framework.

## 1. Introduction

The genus *Flavivirus* includes important human zoonotic pathogens such as dengue virus (DENV), yellow fever virus (YFV), West Nile virus (WNV), tick-borne encephalitis virus (TBEV), and Japanese encephalitis virus (JEV). Like WNV, other flaviviruses, such as Usutu virus (USUV) and Bagaza virus (BAGV), can cause severe disease in animals. They are arboviruses, and therefore are transmitted from an infectious to a susceptible vertebrate host via hematophagous arthropod vectors, such as mosquitoes or ticks.

In recent years, the areas in Europe reporting flavivirus infections and specifically WNV, USUV, or TBEV have significantly increased [1]. Moreover, many of these viruses are currently endemic in several areas of the continent. Expansion and endemicity increase spatial and temporal overlapping of flaviviruses in Europe, which in turn poses significant challenges for surveillance and control [2]. Given the trends in climate warming and land-use changes, as well as the increase of human travel and the intensification of the trade of animals and goods, the situation is likely to become even more complex in the future [3].

Cases of WNV infection in horses and humans have been reported in Europe for decades. Before 2004, all outbreaks had been caused by WNV lineage 1; however, in 2004, WNV lineage 2 was detected, both in a northern goshawk (*Accipiter gentilis*) with neurological symptoms in Hungary [4] and in two human patients from the Rostov region in western Russia [5]. In the following years, WNV lineage 2 spread within Eastern and Central/Southern Europe, where the virus has remained endemic, causing hundreds of cases in humans and horses [6]. In Spain, cases of WNV in horses and humans caused by a WNV lineage 1 strain have been reported in the south of the country since 2010 [7]. In September 2017, WNV lineage 2 was detected in Catalonia (northeastern Spain) in a northern goshawk, evidencing WNV lineage 2 spread to Western Europe [8]. In 2018, Europe experienced the largest number of WNV infections to date (with more than 2000 human cases), and other large-scale epidemics are to be expected in the future [9]. More recently, WNV expansion in Europe continued, and the first human cases were reported in Germany and the Netherlands in 2019 and 2020, respectively [10]. In Spain, the 2020 season resulted in an unprecedented increase in the number of human cases with 77 cases reported, in contrast to the six cases reported in the previous two decades [11].

In Europe, USUV was first detected in Austria in 2001 [12], although retrospectively the virus was identified from Eurasian blackbirds (*Turdus merula*) found dead in the Tuscany region (Italy) in 1996 [13]. In 2006, USUV was detected in a pool of *Culex pipiens* mosquitoes captured in the Delta del Llobregat, a wetland near the city of Barcelona [14]. Its genome showed the closest relationship to an USUV strain isolated in South Africa in 1959 [15]. A related strain was isolated from a pool of *Culex perexiguus* mosquitoes in southern Spain in 2009 [16]. Neither strain detected in Spain seemed highly virulent for birds, and no disease was documented, while the USUV strains reported in Italy, Austria, Hungary, Switzerland, Germany, and the Czech Republic were associated with epizootics in Passeriformes and Strigiformes [15,17]. In 2012, a USUV strain closely related to the one reported in Central Europe was detected in two migratory song thrushes (*Turdus philomelos*) with neurological signs in southern Spain [18]. Up to 2019, 46 human cases of USUV infection had been recorded in Europe, and although most of them were accidentally detected in asymptomatic blood donors, USUV is now acknowledged as having the potential to cause severe neurological disease in humans [17].

In 2010, Bagaza virus (BAGV), a flavivirus previously detected in sub-Saharan Africa, India, and Israel, was identified after an episode of high mortality in partridges (*Alectoris rufa*) and common pheasants (*Phasianus colchicus*) in Cadiz (southwestern Spain) [19]. Circulation of BAGV was confirmed the following season in the same area, suggesting virus overwintering [20]. In Catalonia, circulation of BAGV was detected in chickens by serology in the south of the region in January 2018 [8], and in Eurasian magpies (*Pica pica*) sampled in the west of the region in June 2018 [21]. 

Among the flaviviruses present in Europe, TBEV is responsible for tick-borne encephalitis (TBE), considered the most important viral tick-borne disease in Europe [22]. It is a notifiable disease in the European Union since 2012, and between 2012 and 2016, 12,500 TBE human cases were reported in 23 of the 28 EU countries plus Iceland and Norway [23]. No autochthonous human cases were detected in Spain during that period or in 2017–2019, the last years for which data is available [24]. In recent years, distribution of TBEV in Europe seems to have expanded, with new areas affected and an increase of the number of cases reported in several countries. For example, three autochthonous human cases of TBE were detected in 2017 and 2018 in rural areas of central France not previously affected [25]. Migrating birds may carry ticks infected with TBEV and therefore contribute to the spread of the virus to new areas [26].

While molecular diagnosis is essential for the confirmation of clinical cases of WNV, serological assays are a very useful tool for surveillance (i.e., determining the prevalence of infection). However, the antigenic similarities among flaviviruses frequently result in cross-reactions, which compromises the serological diagnosis of flavivirus infections [27]. Therefore, the serological results need to be confirmed by comparative neutralization tests. The neutralization tests must be carried out against a panel of flaviviruses known to circulate in the area, for which prior knowledge of viral diversity is critical. Correct diagnosis of flaviviruses is further complicated when they belong to the same serocomplex, such as in the cases of WNV and USUV, as cross-neutralization may occur [27]. Due to the spatial and temporal overlapping of flaviviruses in Europe, their differentiation by diagnostic tests is essential for surveillance and control [2]. Co-circulation of WNV and USUV in Europe has been evaluated, but at country level (i.e., countries where circulation of these viruses has been detected) [28,29]. However, to assess whether they have a similar geographical distribution, and therefore whether their ecological requirements are likely to be the same, a much lower spatial aggregation, preferably point data, is required.

Since 2007, an integrated ecological surveillance program for WNV has been implemented in Catalonia, which includes testing of horses and wild birds [30]. Through that program, flaviviruses not identified as WNV have been consistently identified in both horses and birds from Catalonia [8,21,30]. With the aim of better characterizing the flaviviruses circulating in Catalonia between 2010 and 2019, serum samples from birds and horses positive by panflavivirus competition ELISA (cELISA) were further analyzed by microneutralization test (MNT) against different flaviviruses. Samples from horses were tested for the presence of antibodies against WNV, USUV, and TBEV, while samples from birds were tested for the presence of antibodies against WNV, USUV, and BAGV. Furthermore, we also evaluated the spatial distribution of those flaviviruses and assessed whether there was any potential geographical aggregation (i.e., clustering). The ultimate goal of the study is to improve the surveillance and control of flaviviruses in Catalonia.

## 2. Materials and Methods

### 2.1. Laboratory Analyses

#### 2.1.1. Birds

Of 3791 serum samples collected from birds between 2010 and 2019 in Catalonia within the WNV Surveillance Program, 380 tested positive by panflavivirus cELISA (IDvet), and of them, 205 had enough serum to be tested by micro-neutralization test (MNT) at the CISA laboratory in Madrid. Birds’ samples originated from a variety of sources, which comprised the wildlife recovery centers of Catalonia where both healthy wild birds and wild birds suspected to be infected by WNV were sampled. Other samples were obtained through specific surveys in certain species (e.g., Eurasian magpies or pigeons), sentinel chickens, a cross-sectional survey in chicken farms throughout Catalonia, or surveys in chicken farms in the proximity of confirmed WNV outbreaks. They included birds from 25 species, primarily Eurasian magpies with 77 samples and chickens (*Gallus gallus*) with 49. 

To measure neutralizing antibody responses against different flaviviruses, MNTs were performed in parallel against WNV (strain E101), USUV (SAAR-1776), and BAGV (Spain/RLP-Hcc1/2010) (Genbank accession nos. AF260968, AY453412 and KR108244, respectively). MNT was performed following the protocol previously described by Llorente and collaborators (2019) [31]. Briefly, twofold serial dilutions of test sera were added in 96-well micro titer plates and mixed with an equal volume containing 100 TCID_50_ of virus, resulting in final serum dilutions starting at 1:10. After 1 h incubation at 37 °C, a suspension of approximately 10^4^ Vero cells was added to the wells, and the plates were incubated at 37 °C and 5% CO_2_ for 4–5 days for WNV and 5–6 days for USUV and BAGV. The presence or absence of complete cytopathic effect (CPE) was determined in each well by reading the plates under an inverted light microscope. Neutralization titers were assigned based on the highest dilution of each serum able to abolish CPE and thus neutralize the infection. Samples were classified as positive for WNV, USUV, or BAGV infection if positive for that virus by MNT (titer ≥1:10) and negative for the remaining viruses, or when the titer for a virus (WNV, USUV, or BAGV) was at least 4 times higher than for the remaining viruses. Otherwise, the sample was classified as infected by an “undetermined flavivirus”.

#### 2.1.2. Horses

Of 1856 serum samples collected from horses and tested by panflavivirus cELISA (IDvet) between 2010 and 2019 in Catalonia within the WNV Surveillance Program, 182 tested positive, and of them, 164 had enough serum to be tested by MNT at the ANSES laboratory (Maisons-Alfort, France). Horses’ samples originated also from different sources, which comprised the Equine Veterinary Hospital where both healthy horses and horses suspected to be infected by WNV were sampled. Other samples were obtained through surveys carried out in horses’ slaughterhouses, a cross-sectional survey in horses’ holdings throughout Catalonia, or surveys in horses’ holdings in the proximity of confirmed WNV outbreaks. All those samples were investigated through MNT for the detection of specific neutralizing antibodies against WNV, USUV, and TBEV using WNV (strain IS-98- ST1, Genbank accession no. AF481864.1, provided by P. Desprès, IPP), USUV (strain France 2018, Genbank IF MT863562.1), and TBEV (strain Hypr, Genbank accession no. U39292.1), following the protocol described by Beck and collaborators (2015) [32]. Samples were classified as positive for WNV, USUV, or TBEV infection if positive for that virus by MNT and negative for the remaining viruses, or when the titer for a virus (WNV, USUV or TBEV) was at least 4 times higher than the titers for the remaining viruses. Otherwise, the sample was classified as infected by an “undetermined flavivirus”.

The distribution of samples in birds and horses collected within the WNV Surveillance Program in Catalonia between 2010 and 2019 is shown in the Supplementary Figure S1.

### 2.2. Spatial Analyses

To evaluate the geographical spread of the different flaviviruses in Catalonia, we estimated the number of geographical units (i.e., 42 comarques of Catalonia) in which each particular flavivirus was detected.

We also assessed the potential geographical aggregation (clustering) of flaviviruses in Catalonia using a purely spatial Bernoulli model implemented in SaTScan [33]. It allows evaluating whether there is any area where the distribution of a given flavivirus is different from the rest of the study region. A purely spatial rather than a spatio-temporal analysis was carried out because of the impossibility to determine the date of infection of the animals. For the Bernoulli model, two categories, WNV-positive samples and USUV-positive samples were used, and both, areas of high and low rates were scanned. Only data from horses and chickens were considered to reduce the risk of the animals having been infected in a location different from where they were sampled. To avoid the analysis being conditioned by having several infected animals from the same farm (because of within-farm analyses after detection), we chose farms as units and used unique locations for a given flavivirus. As a result, 58 locations (44 WNV and 14 USUV) were included in the analysis, nine of which corresponded to chickens and 49 to horses.

## 3. Results

Overall, 10.0% (380/3791) of the samples from birds and 9.8% (182/1856) of the samples from horses collected between 2010 and 2019 in Catalonia, tested positive by cELISA.

### 3.1. Flaviviruses in Birds

**Figure 1:**
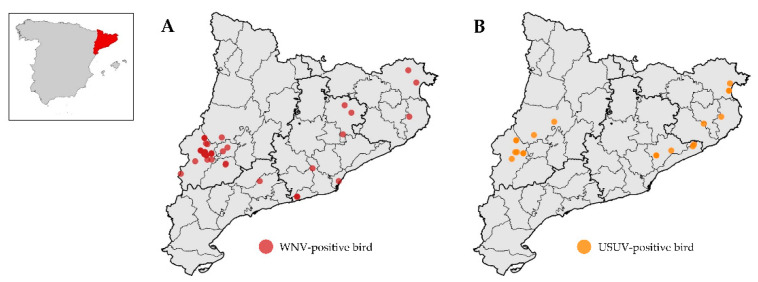
Distribution of WNV-positive birds (**A**) and USUV-positive birds (**B**). The geographical units within Catalonia are the 42 comarques. On the left, location of Catalonia (in red) within Spain.

Of the 205 serum samples from birds tested by MNT, 118 showed specific antibodies for WNV, 19 for USUV, and 68 were classified as undetermined flavivirus. None were classified as BAGV infection. The geographical distribution of the birds positive for WNV and USUV is shown in Figure 1. WNV and USUV positive birds were detected from as early as 2010 and throughout the years of study.

The 118 birds infected by WNV in Catalonia belonged to 14 species and six families. Of those 14 species, six were from the family Accipitridae. The 19 birds infected by USUV in Catalonia belonged to 10 species and nine families. Of those 19 birds, six were chickens. The 68 birds exposed to undetermined flavivirus belonged to 18 species and 10 families. Of them, 17 were raptors (families Accipitridae, Strigidae, and Tytonidae), and 20 were Eurasian magpies. All these results, including the MNT titers in positive birds, are shown in Table 1.

### 3.2. Flaviviruses in Horses

Of the 164 sera from horses tested by MNT, 92 were positive for WNV, 11 for USUV, four positive for TBEV, and 57 were classified as infected by an undetermined flavivirus. The distribution of MNT titers in horses infected by USUV, WNV, and TBEV is shown in Table 2.

**Figure 2:**
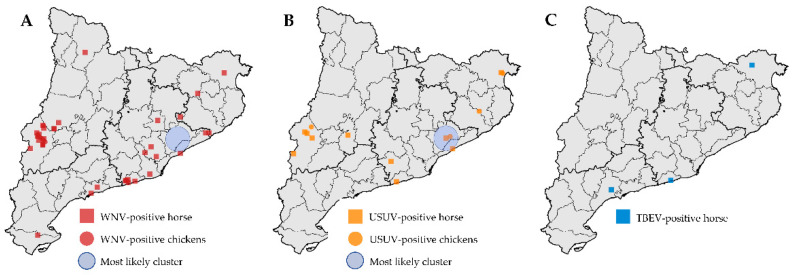
Distribution of WNV-positive samples (**A**) and USUV-positive samples (**B**) used for the purely spatial Bernoulli model implemented in SaTScan for WNV and USUV, which included horses and chickens. Distribution of TBEV-positive samples in horses (**C**).

Two of the samples positive for TBEV were collected in 2016 in northeastern Catalonia from the same horse with 10 days difference, and the other two from horses sampled in 2013 from different locations in southeastern Catalonia (Figure 2C). Further investigations revealed that all the animals had stayed at some point outside Catalonia, so the possibility of imported infections could not be totally excluded.

### 3.3. Spatial Analyses

Considering both birds and horses, USUV had a more restricted geographical distribution limited to the central-western and northeastern areas of Catalonia, while WNV was detected in the areas where USUV was reported, but also in central and southern Catalonia (Figure 1 and Figure 2). In fact, USUV was detected only in 10 out of the 42 comarques of Catalonia and WNV in 18 (Figure 1 and Figure 2). 

The purely spatial Bernoulli model indicated that there were no statistically significant clusters for WNV nor USUV in Catalonia. The most likely cluster was located in central-eastern Catalonia and had a radius of 13.1 km (Figure 2). It had three cases of USUV although 0.7 was expected assuming random distribution, which represents 5.0 times more than expected (Table 3). It had no cases of WNV although 2.3 were expected. However, the most likely cluster was not statistically significant (*p* = 0.12). All the remaining potential clusters evaluated were far from statistical significance.

## 4. Discussion

In our study, 10.0% of the birds tested positive by cELISA indicating the exposure to flavivirus in the bird population of Catalonia. In a serosurvey carried out in wild birds admitted to rehabilitation centers in Extremadura, western Spain, between 2017 and 2019, of the 384 animals tested, 120 (31.3%) were positive by ELISA (INgezim West Nile Compac, Ingenasa) [34]. Such high prevalence compared to our study may be explained by differences in the true prevalence of flaviviruses between areas, in the species selected or in the ELISA test used. 

Although birds with specific antibodies against WNV were detected from as early as 2010, only 13 of the 118 positive birds (11%) were detected before the molecular confirmation of WNV circulation in Catalonia in 2017 [8]. Moreover, none of those 13 birds belonged to resident species, even though 510 resident birds had been tested between 2010 and 2016. In total, 11 of the 13 positive birds were raptors (six short-toed snake eagles (*Circaetus gallicus*), three common kestrels (*Falco tinnunculus*), and two European honey buzzards (*Pernis apivorus*)), while the two other birds were white storks (*Ciconia ciconia*). These results are consistent with previous observations of very low or no circulation of WNV in Catalonia before 2017 [30].

Bird sera positive for USUV were consistently detected throughout the period of study (2010–2019) and were not linked to any episode of mortality in birds. So far, the only USUV strain isolated in Catalonia was detected in a pool of *C. pipiens* near the city of Barcelona in 2006 [14] and seemed to be a strain less pathogenic for birds than others circulating in Central Europe [15]. Whereas the genetic diversity of the USUV strains in Central Europe seem to be the result of a single introduction from Africa and the establishment of an endemic cycle, the African strains detected so far in Spain appear the result of repeated introductions by migratory birds from different geographic origins [35]. Further studies would be needed to characterize the USUV strains present in Catalonia and to determine their potential origin and pathogenicity. Positivity to WNV and USUV in birds have also been reported in other regions of Spain, such as in Andalusia (in the south) [20,36] and in Extremadura (in the west) [34].

Of the 19 birds with specific antibodies against USUV, eight were MNT-positive for USUV and negative for WNV and BAGV. Therefore, under a testing scheme in which cELISA-positive birds are not confirmed by MNT against USUV, those eight USUV positive birds would have not been identified. Furthermore, the remaining 11 birds positive for USUV by MNT were also positive for WNV and two also for BAGV, so if not confirmed by MNT for USUV, samples would have been misclassified as WNV or BAGV. Moreover, Llorente and collaborators [31] showed that cross-reactions between WNV and USUV in red-legged partridges were not symmetric, as WNV cross-reaction in USUV-infected birds was higher than USUV cross-reaction in WNV-infected birds. As a result, while all WNV-infected birds were correctly identified, none of the 10 USUV-infected birds was correctly classified (their MNT titers against USUV were less than fourfold higher than against WNV). Therefore, even when cELISA-positive samples are tested for both WNV and USUV, as in the case of the current study, USUV-infections are likely to be underestimated.

There were several species and families of birds infected by USUV in Catalonia, the majority of which had been reported in other countries [17,37]. Only one species had not been previously identified as infected by USUV, the bearded vulture (*Gypaetus barbatus*), a near threatened raptor species. Chvala and collaborators [38] concluded that chickens were unlikely to be useful as sentinels for USUV after finding that only one out of the 10 chickens inoculated with the Vienna 2001-blackbird (939/01) USUV strain developed antibodies. In contrast, in Catalonia, chickens represented 32% (6/19) of the birds positive for USUV (and with MNT titers as high as 1/640) despite representing only 24% (49/205) of birds tested for flaviviruses, indicating that they may be good sentinels for USUV. Furthermore, chickens also proved useful for WNV surveillance as they were 29% (34/118) of the birds positive for WNV (and with MNT titers as high as 1/1280) despite representing only 24% of birds tested, in agreement with previous findings [39].

Despite a significantly higher number of WNV-positive birds compared to USUV (118 vs. 19 birds, respectively), there appears to be much less variation in the types of birds infected (six families for WNV and nine for USUV). Roiz and collaborators [40] found a positive correlation between the prevalence of USUV in *Culex perexiguus* and the richness of Passeriformes, which explained more variance than any other climatic, landscape, vector, or host variable. In our study, we observed a wide variation in the species infected by USUV, but those species belonged to several different bird orders, not just Passeriformes.

Of the bird species affected by flaviviruses, a significant proportion were raptors, mainly from the family Accipitridae. That is likely explained by the predation of infected birds by raptors [41] acquiring flavivirus infection by the oral route. Interestingly, infection of raptors was more evident for WNV than for USUV. 

BAGV was detected in resident birds (chickens and magpies) of Catalonia in 2018 [8,21], but our results show that BAGV infections in the region do not seem very common. Even though we detected positivity to BAGV in 53 of the 148 birds tested, with titers ranging from 1/5 to 1/80, in all cases titers against WNV or USUV were even higher, so none were classified as BAGV-positive. Considering that BAGV belongs to a different serocomplex, a sample positive by MNT for both BAGV and other flaviviruses, in particular when BAGV titers are high, may potentially be the result of co-infections or sequential infections. However, Llorente and collaborators [31] found that in BAGV-infected birds, cross-reactions with other flaviviruses (mainly with WNV, but also with USUV) are common, and therefore some true BAGV-infected animals in our study may have been missed. 

In a cross-sectional survey carried out in horses throughout Andalusia (southern Spain) after the 2010 epidemic, 10.6% (54/510) of the animals tested positive by cELISA (INgezim West Nile Compac, Ingenasa) [42], quite similar to the 9.8% detected in our study despite epidemiological differences between the areas or in the ELISA test used.

We found that WNV, and to a lesser extent USUV, have been circulating in horses from different areas of Catalonia between 2010 and 2019. In 2018, the first three WNV outbreaks in horses were officially reported in Catalonia in the central-eastern area [21]. Before that, no positivity to IgM ELISA had been detected. Our results show that at least from as early as 2011, WNV positivity by MNT was found in horses from different areas of Catalonia, although the unequivocal circulation of the virus could not be proven until 2017, when WNV genome was detected in a northern goshawk by PCR [8]. USUV-positivity had not been reported previously in horses from Catalonia.

TBEV-positivity was also sporadically detected in horses from some areas of Catalonia. In a study carried out in southeastern Sweden, of the 1155 ticks collected from 13,260 screened birds, 98% were *Ixodes ricinus* [26]. Six birds carried TBEV-infected ticks, one tree pipit (*Anthus trivialis*), one song thrush (*Turdus philomelos*), one common redstart (*Phoenicurus phoenicurus*), and three European robins (*Erithacus rubecula*). All these are ground-feeding passerine species that migrate from northern and central Europe, where TBE is endemic, to Catalonia [43], which could explain the mechanism of introduction of the virus. No further TBEV-positivity was detected in the affected areas in subsequent years despite intense sampling in horses in some of those locations, which would seem to indicate the failure to establish endemic foci. A longitudinal study conducted in an endemic focus of TBEV in eastern France over seven years showed that transmission is very sensitive to some abiotic and biotic factors [44]. In fact, the variations in densities of larvae and nymphs because of special meteorological conditions, seemed to result in the endemic fadeout of TBEV in the area, which would explain the low incidence in humans in that region. According to the current known distribution, *Ixodes ricinus* ticks, the main vector of TBE in Europe, is not present in Catalonia [45]. Studies on the possible presence of *Ixodes ricinus* ticks in Catalonia are needed to evaluate the risk of TBE. In a recent study, Crimean-Congo hemorrhagic fever virus antibodies were detected in Catalonia despite apparent absence of *Hyalomma marginatum* and *H. lusitanicum* ticks, the main vectors in Europe [46]. Specific neutralizing antibodies against TBEV have been detected in dogs from western and southern Spain [47], and in a horse from the island of Majorca [48]. Camino and collaborators [49] reported positivity against TBEV in horses from several areas in Spain including one horse from Catalonia sampled in 2016. However, samples had not been tested against USUV because probability of USUV infection was considered minimal, while our results show that USUV infections in horses have occurred in Catalonia for many years. Even though TBEV and USUV belong to different serocomplexes and therefore the risk of cross-reaction is lower [50], this possibility cannot be ruled out. Moreover, studies of flavivirus detection in horses, in particular TBEV, should consider the possibility of imported infections.

A significant proportion of the samples positive by cELISA and tested by MNT were classified as undetermined flavivirus, 33.2% (68/205) in birds and 34.8% (57/164) in horses. A likely explanation is that the cELISA is more sensitive than the MNT, at least for WNV, and therefore low antibody titers may be detected by ELISA but not by MNT [51]. Another possibility would be the infection by a flavivirus different from those evaluated by MNT, which may cross-react with the antigen employed in the cELISA. A Meaban-Like virus, another flavivirus, was detected in the northern coast of Catalonia in yellow-legged gulls (*Larus michahellis*) [52]. Louping ill virus, another zoonotic flavivirus antigenically related to TBEV and also transmitted by *I. ricinus*, has been sporadically reported in areas of northern Spain [53]. Finally, the possibility of co-infections or sequential infections of different flaviviruses may also explain some of the results obtained in the samples classified as undetermined flaviviruses. 

The high degree of spatial overlapping observed between WNV and USUV infections seems to indicate that their ecological determinants are essentially similar. Indeed, they seem to share the main vectors and reservoirs, *Culex* spp. mosquitoes and passerine birds, respectively [54]. However, the fact that there are areas where WNV is present but USUV is absent, may reflect differences in some ecological aspects. For example, the temperature requirements for vector competence for WNV and USUV in *C. pipiens* seem to be different [55]. Furthermore, the results of the spatial analysis indicated that there were no areas where the transmission of WNV or USUV were significantly higher or lower than the rest of Catalonia. The most likely cluster, located in central-eastern Catalonia, had an increased risk of USUV and a reduced risk of WNV, but differences were not statistically significant.

For the purpose of our study, which was the identification of different flaviviruses circulating in Catalonia, the screening test we used (cELISA, IDvet), which is able to detect infections by a large range of flaviviruses [31], was suitable. In a different epidemiological context, such as if the objective was the identification of WNV infections exclusively, a test with a higher specificity [31], may be preferred. In any case, the molecular diagnosis of flaviviruses is still necessary for the identification of the etiologic agent. In fact, during the period study the two northern goshawks positive for WNV by RT-qPCR in 2017 [8] were the only samples confirmed by molecular techniques.

There are several sources of bias in the selection of animals for the study that need to be acknowledged. In the case of birds, some species were over-represented either because they were useful for WNV surveillance (e.g., chickens or magpies), because they have ecological value and were frequently found in wildlife recovery centers (e.g., raptors) or because samples were commonly collected within other surveillance programs (e.g., Columbidae for Newcastle disease and Anatidae for avian influenza). In horses, the distribution of samples may be influenced by the proximity to the Equine Veterinary Hospital. Finally, some regions, mainly the areas where WNV circulation was detected, were also over-represented as a result of the specific surveys implemented there.

## 5. Conclusions

Given the widespread distribution of WNV and USUV in Catalonia, MNT for both viruses should be included as a routine technique for the correct diagnosis of flavivirus infections in the region. Failure to do so results in both under-detection and misclassification. Considering the sporadic detection of TBEV, further studies of the risk of introduction and establishment would be needed.

So far, no human infections by zoonotic flaviviruses such as WNV, USUV, or TBEV have been reported in Catalonia. However, considering that these viruses have been circulating for some time in different areas of Catalonia means that they need to be kept under surveillance, and the most efficient way to do that is within a One Health framework. That includes raising awareness among medical practitioners (clinicians), to consider these infections in the differential diagnosis of patients with compatible clinical symptoms.

## Figures and Tables

**Table 1:** Detection of antibodies against flaviviruses in sera from different bird species by MNT. In brackets MNT titer (or range if more than one positive sample).

Scientific Name	Common Name	Family	Order	Undetermined Flavivirus	USUV	WNV	Total
*Aquila fasciata*	Bonelli’s eagle	Accipitridae	Accipitriformes			1 (1/40)	1
*Buteo buteo*	Common buzzard	Accipitridae	Accipitriformes	3	1 (1/10)	1 (1/20)	5
*Circaetus gallicus*	Short-toed snake eagle	Accipitridae	Accipitriformes	6		6 (1/20 to 1/160)	12
*Circus aeruginosus*	Western marsh harrier	Accipitridae	Accipitriformes	1			1
*Gypaetus barbatus*	Bearded vulture	Accipitridae	Accipitriformes		1 (1/10)	5 (1/160 to 1/1280)	6
*Gyps fulvus*	Griffon vulture	Accipitridae	Accipitriformes	1			1
*Milvus migrans*	Black kite	Accipitridae	Accipitriformes	1			1
*Milvus milvus*	Red kite	Accipitridae	Accipitriformes			1 (1/20)	1
*Pernis apivorus*	European honey buzzard	Accipitridae	Accipitriformes	1		3 (1/40 to 1/80)	4
*Anser anser*	Greylag goose	Anatidae	Anseriformes		1 (1/160)		1
*Larus michahellis*	Yellow-legged gull	Laridae	Charadriiformes	4			4
*Ciconia ciconia*	White stork	Ciconiidae	Ciconiiformes	4	2 (1/20 to 1/640)	4 (1/20 to 1/640)	10
*Columba livia*	Rock pigeon	Columbidae	Columbiformes	2	2 (1/80)	1 (1/40)	5
*Columba palumbus*	Common wood pigeon	Columbidae	Columbiformes			1 (1/10)	1
*Streptopelia decaocto*	Eurasian collared dove	Columbidae	Columbiformes	1			1
*Falco tinnunculus*	Common kestrel	Falconidae	Falconiformes	2	1 (1/320)	4 (1/20 to 1/160)	7
*Alectoris rufa*	Red-legged partridge	Phasianidae	Galliformes			1 (1/160)	1
*Gallus gallus*	Chicken	Phasianidae	Galliformes	9	6 (1/10 to 1/640)	34 (1/10 to 1/1280)	49
*Corvus corax*	Common raven	Corvidae	Passeriformes	3		1 (1/10)	4
*Pica pica*	Eurasian magpie	Corvidae	Passeriformes	20	2 (1/10 to 1/160)	55 (1/20 to 1/640)	77
*Ardea cinerea*	Grey heron	Ardeidae	Pelecaniformes	3	2 (1/80 to 1/160)		5
*Asio otus*	Long-eared owl	Strigidae	Strigiformes	1			1
*Bubo bubo*	Eurasian eagle-owl	Strigidae	Strigiformes		1 (1/40)		1
*Strix aluco*	Brown owl	Strigidae	Strigiformes	4			4
*Tyto alba*	Western barn owl	Tytonidae	Strigiformes	2			2
Total				68	19	118	205

**Table 2:** Distribution of MNT titers in samples from horses infected by USUV, WNV, and TBEV.

Titers	USUV	WNV	TBEV
1/10	5	5	
1/20	1	15	
1/40	4	25	1
1/80	1	21	1
1/160		15	
1/320		11	2
Total	11	92	4

**Table 3:** Results of the most likely cluster in the purely spatial Bernoulli model implemented in SaTScan.

	Number of Cases	Expected Cases	Relative Risk	*p*-Value
USUV	3	0.72	5.0	0.12
WNV	0	2.28	0.0	

## Data Availability

Data not available because of legal (confidentiality) issues.

## Supplementary Materials

The following are available online at https://www.mdpi.com/article/10.3390/v13122404/s1, Figure S1: Distribution of samples collected within the WNV Surveillance Program in Catalonia between 2010 and 2019: 3791 serum samples from birds (A); and 1856 samples from horses (B). Samples of birds and horses in the map are represented with transparency; so that darker greys indicate overlapping of samples.
