# Supplementary material for: Widespread Circulation of Flaviviruses in Horses and Birds in Northeastern Spain (Catalonia) between 2010 and 2019
Source: Viruses. 2021 Nov 30;13(12):2404. doi: 10.3390/v13122404 (PMC8708358; doi:10.3390/v13122404)
Supplement: Supplementary file 1 [file viruses-13-02404-s001.zip › viruses-1474119-supplementary.pdf]

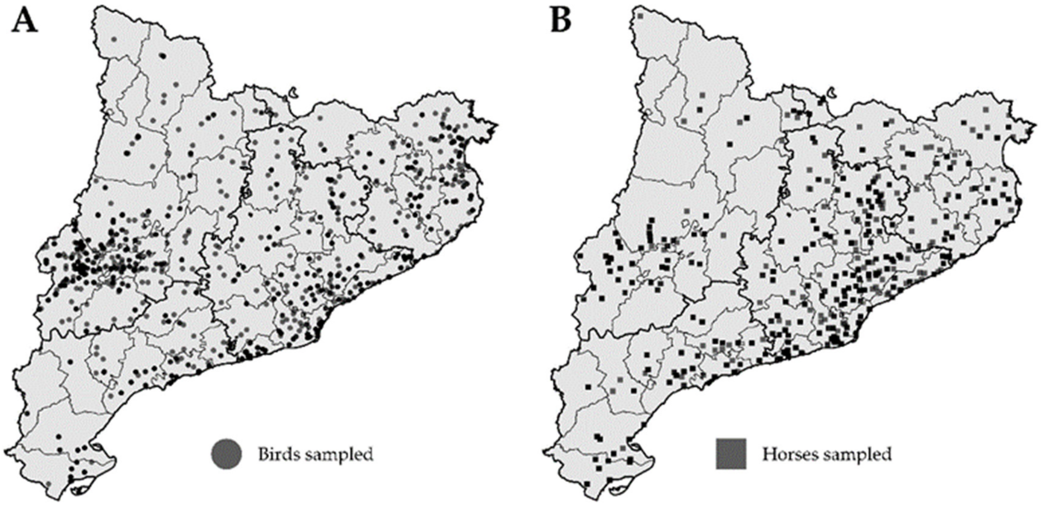

Figure S1: Distribution of samples collected within the WNV Surveillance Program in Catalonia between 2010 and 2019: 3791 serum samples from birds (A); and 1856 samples from horses (B). Samples of birds and horses in the map are represented with transparency; so that darker greys indicate overlapping of samples
